# The Relationship Between Anti-Transgender Experiences (A Proxy for Minority Stress) and Heavy Alcohol Use Among Transgender Adults

**DOI:** 10.3390/bs15030248

**Published:** 2025-02-21

**Authors:** Hugh Klein, Thomas Alex Washington

**Affiliations:** 1 Kensington Research Institute, Silver Spring, MD 20910, USA; 2 School of Social Work, California State University, Long Beach, CA 90840, USA; alex.washington@csulb.edu

**Keywords:** transgender, heavy drinking, minority stress, anti-transgender experiences

## Abstract

Purpose: Although several studies have shown a relationship between anti-transgender experiences and binge drinking and/or hazardous drinking, very little research has examined how these experiences relate to heavy drinking. That is the focus of this study. Methods: This paper uses data from the 2015 National Transgender Survey, and is based on a sample of 17,367 transgender adults residing in the United States. Analyses compare three groups: current “regular” drinkers (drank at least some alcohol during the previous month but no days consuming five or more drinks) (*n* = 10,496), binge drinkers (consumption of five or more drinks on at least one occasion during the previous month) (*n* = 4977), and heavy drinkers (five or more drinks per day on five or more days during the previous month) (*n* = 1894). The paper focuses on how anti-transgender experiences with harassment, discrimination, and/or violence (a 20-item scale measure, Cronbach’s alpha = 0.76) are related to people’s classification as current drinkers versus binge drinkers versus heavy drinkers. Results: 13.8% of the participants met the criteria for heavy drinking; 26.4% more were classified as binge drinkers. The more anti-transgender experiences people had, the more likely they were to engage in heavy drinking. Multivariate analyses revealed that this relationship was a robust one, holding up even when numerous other potentially confounding control measures were included in the analyses. Conclusions: Anti-transgender experiences are a strong predictor of heavy drinking. This type of minority stressor is an important consideration when understanding what leads many transgender individuals to become heavy drinkers.

## 1. Introduction

The term “minority stress” is thought to date back to 1981 (Brooks, 1981), when it was first applied to the study of how exposure to cultural, social, and socioeconomic stressors among certain socially disadvantaged groups (in the original work, this was women who self-identified as lesbians) led members of these groups to experience various types of psychological problems/stressors and physical symptoms resulting from experiencing those symptoms. A little more than a decade later, this concept was expanded by Meyer (1995) to include gay men as one of the affected groups, and by examining three specific types of minority stressors, to understand how those stressors affected a variety of mental health outcomes. In subsequent years, the construct of minority stress has been transformed into a full-fledged theory to help scientists understand the myriad negative consequences that members of sexual minority groups (e.g., gay men, bisexual persons, transgender persons, etc.), members of racial minority groups, and a host of other social “out groups” experience when they face discrimination, harassment, and/or violence because of their identity as a member of some type of minority group.

As such, a fair amount of minority stress-focused research has been conducted with transgender populations, relatively consistently finding that experiencing greater amounts of minority stress is associated with a wide variety of adverse health outcomes. Included among these are: increased risk for cardiovascular disease (Poteat et al., 2021), diminished body satisfaction (Klemmer et al., 2021; Mitchell et al., 2021; Muratore et al., 2022), elevated risk of engaging in unhealthy eating practices (Mitchell et al., 2021; Muratore et al., 2022), increased risk of experiencing anxiety (Dolezal et al., 2023; McLemore, 2018; Puckett et al., 2023a, 2023b), greater risk of experiencing depression or incurring higher levels of depressive symptomatology (Hoy-Ellis & Fredriksen-Goldsen, 2017; McLemore, 2018; Pellicane & Ciesla, 2022; Puckett et al., 2023a, 2023b), diminished levels of self-esteem (Austin & Goodman, 2017), heightened sense of hopelessness (Parr & Howe, 2019; Tebbe et al., 2022), increased odds of engaging in substance misuse/abuse (Puckett et al., 2023b; Wolford-Clevenger et al., 2021).

Along these lines, the concept of *allostatic load* has been introduced (McEwen & Stellar, 1993) and used to explain the nexus of transgender persons’ experiences with anti-transgender discrimination, harassment, and violence and these adverse outcomes. *Allostatic load* refers to the ‘wear and tear’ on the body that accumulates after someone has been exposed to repeated and/or chronic illness (McEwen & Stellar, 1993). In recognition of the strength of the mind–body connection, the construct of allostatic load has been expanded beyond its original focus on the mere physiological impact of repeated stressful exposures on the body and now encompasses the emotional, psychological, and social impact of such exposures (see, for example, Juster et al., 2010; Kristenson et al., 2004). Research undertaken in recent years has demonstrated—fairly consistently—that exposure to minority stressors elevates transgender persons’ allostatic load^1^ (DuBois & Juster, 2022; Ferina, 2024), and, in turn, elevated allostatic load has been shown to be associated with a greater risk of substance use/abuse (Guidi et al., 2021).

In light of the research focusing on minority stress and adverse outcomes and the research focusing on allostatic load and adverse outcomes, it is not surprising that a number of studies have examined substance misuse/abuse, particularly alcohol misuse/abuse, among transgender individuals. Almost all of these studies have reported ***much*** higher-than-average rates of drinking-related problems and unhealthy drinking practices among transgender persons than among other groups in the population at large. For example, Puckett et al. (2023b) found that 35.4% of the 181 transgender and gender-diverse people in their study reported *hazardous drinking levels* during the previous six months. Fahey et al. (2023) conducted a review of the literature examining binge drinking among transgender and gender-diverse youth aged 10–24. They found that anywhere from 11 to 59% reported *binge drinking*. Of relevance to the present study, Fahey et al. (2023) found that, in general, increased exposure to victimization was associated with an increased risk of binge drinking. As another example, Tran et al. (2023) conducted a study of 2324 transgender and nonbinary Canadians. They discovered that 16.9% of their study participants reported past-year *hazardous alcohol drinking*. Moreover, the amount of lifetime anti-transgender discrimination (OR = 1.37) and the amount of lifetime major discrimination (OR = 1.69) were both associated with an increased risk of hazardous alcohol drinking. In a study comparing transgender (*n* = 58) and nontransgender (*n* = 482) active-duty members of the military, transgender persons were 1.48 times more likely to engage in *hazardous drinking* (Holloway et al., 2021). An even greater prevalence was reported by Reisner and Hughto (2019). Their study of 452 Massachusetts adults who self-identified as transgender or gender-nonconforming found a 40.9% *hazardous drinking* rate. Kcomt et al. (2020) used data from the 2015 National Transgender Survey and examined both binge drinking and frequent binge drinking. They reported that 24.3% of the respondents had engaged in *binge drinking* during the previous month, with more than one-third of those individuals qualifying as *frequent binge drinkers*. Kcomt et al. (2020) went on to point out that antitransgender experiences were associated with an increased risk of engaging both in binge drinking and frequent binge drinking. Also relying on data from the 2015 National Transgender Survey, Klein and Washington (2023a) found that 40.1% of all current drinkers reported past-month *binge drinking*. Experiencing any anti-transgender problems elevated the risk of binge drinking by 48%; experiencing many such problems elevated this risk by 104%. Similarly, a three-site study of 330 transgender adults (Kidd et al., 2019) found that higher levels of “enacted stigma” (i.e., actual anti-transgender experiences, not anti-transgender experiences merely perceived to be based on discrimination) were linked with *persistent risky drinking*. Johnson et al. (2021) reported on a study of 89 American and Canadian transgender and gender-diverse women. They found that anywhere from 39% to 51% of the study participants met the criteria for *hazardous drinking*, depending on whether a laxer or more restrictive definition of hazardous drinking was applied. In their study, the likelihood of engaging in hazardous drinking increased based on the number of symptoms of trauma that people experienced (more symptoms = heightened risk of hazardous drinking). Based on a sample of 297 transgender, nonbinary, and agender adults, Lindley et al. (2021) found that 16.6% met the World Health Organization criteria for *problematic alcohol use*. All four of the categories of anti-transgender experiences studied were related to problematic alcohol use, but the associations were not strong ones. The authors noted that their findings provided support—but not overwhelmingly strong support—for the notion of minority stress having an impact on problematic alcohol use. Staples et al.’s (2018) study of 317 transgender adults aged 18–44 found that 18% reported *hazardous drinking* or *harmful alcohol consumption*, and 16% reported *heavy episodic * drinking (i.e., binge *drinking*). In a qualitative study of 19 African American transgender women from the Washington, DC, and Baltimore areas, Sherman et al. (2022) reported that 73.7% of the respondents reported *misusing alcohol* in response to a violent or traumatic anti-transgender event. Sherman et al. (2022, p. 55) went on to note that participants “used alcohol, tobacco, and other drugs immediately after exposure to violence and beyond to manage stress-related symptoms and unwanted emotions, such as depressed mood, intrusive thoughts, and disordered sleeping”. In one of the only studies identified by the present authors that specifically examined heavy drinking, Horvath et al. (2014) found that, of 1096 American transgender adults in their research, 8.5% reported *regular heavy drinking* and 8.6% reported *binge drinking*.

In writing up the aforementioned studies examining the prevalence of alcohol-related misuse/problems among transgender persons and the role played by anti-transgender experiences in shaping these prevalence rates, the present authors italicized and underlined the key alcohol-related construct under examination to draw attention to one very important point: Almost all of the published studies in this subject area have focused on so-called hazardous drinking or binge drinking, and very few have devoted their focus specifically to heavy drinking. It is important to understand the nuances in the definitional differences amongst these terms. Most of the time, **hazardous drinking** has been defined by having a score in the 8–15 range on a 0–40 scale on the Alcohol Use Disorders Identification Test (AUDIT) (Alcohol Change UK, 2024). Scores in that range typically indicate that the person’s alcohol use behaviors are having various types of adverse effects on his/her/their life, anywhere from less frequently than once per month to perhaps as frequently as once or twice per month. **Binge drinking,** usually has been defined as any event—even an isolated one—in which the drinker consumes four (if female) or five (if male) drinks in a relatively short period of time, typically a few hours (National Institute on Alcohol Abuse and Alcoholism, 2024). **Heavy drinking**, on the other hand, usually has been defined ***either*** as meeting the binge drinking criteria just listed on at least a weekly basis ***or*** as consuming eight or more drinks per week (if female) or fifteen or more drinks per week (if male) ***or*** engaging in binge drinking on five or more days in any given month (National Institute on Alcohol Abuse and Alcoholism, 2024). Heavy drinking is, therefore, more serious than binge drinking (and in most instances, also more serious than hazardous drinking), because it reflects greater periodicity and, usually, greater quantities of alcohol consumption. The near-total absence of research focusing on heavy drinking among transgender persons represents a significant oversight in the scientific literature.

One important way that the present study contributes to the scholarly literature is its specific focus on heavy drinking among transgender adults. It also expands the existing literature by examining the role that one type of minority stressor—namely, anti-transgender experiences with harassment, discrimination, and/or violence—plays in heavy drinking among members of this population. The authors’ hypothesis is that anti-transgender experiences will heighten transgender persons’ likelihood of engaging in heavy drinking. If the data support this hypothesis, then this research will be able to buttress the use of the minority stressors model when trying to understand heavy drinking practices among transgender persons. It should be noted that the present study closely resembles the work by Kcomt et al. (2020) but differs from their work in some key ways. For example, the present research used a similar but substantively different way of measuring antitransgender experiences. (Neither study’s operational definition is necessarily better than the other, as both ways of measuring the construct in question are valid and informative.) Additionally, the present study focuses specifically on heavy drinking, whereas Kcomt et al.’s (2020) work was somewhat more broad in its focus on binge drinking and alcohol misuse, while also reporting on findings specific to frequent binge drinking. The two studies also differ in terms of their use of transition-related milestones and in terms of the variables examined in the multivariate analyses.

## 2. Methods

### 2.1. Data and Procedures

The data for the present research came from the 2015 U.S. Transgender Survey (USTS2015) (James et al., 2016). Data were collected during the summer of 2015 from a total sample of 27,715 transgender persons residing anywhere in the United States or one of its territories, or who were living overseas while serving actively in the U.S. military. At the time it was conducted, it was the largest study of its kind ever having been undertaken to understand transgender persons’ lives. Access to the survey was centralized via a single online portal/website, and all persons completed the survey online. It could be completed via any type of web-enabled device (e.g., computer, tablet, smartphone, etc.) and was available in both English and Spanish language versions.

The questionnaire collected information pertaining to a wide variety of types of harassment, discrimination, and violence that transgender persons may have experienced in a wide variety of settings, such as work, school, public restrooms, public places, governmental offices, while serving in the military, among others. The USTS2015 questionnaire contained some information pertaining to substance use and mental health functioning. It also captured information about various aspects of the transitioning process, including social aspects of transitioning (e.g., divulging information about one’s transgender identity to partners, friends, family members, coworkers, etc.), taking hormone treatments, and various surgical procedures that might be undergone to facilitate gender identity integration. Detailed demographic-type data about each respondent were also collected.

Participants were offered the opportunity to win either a $500 participation grand prize (*n* = 1) or a $250 participation prize (*n* = 2), chosen by random at the end of the data collection period. More than one-third (35.2%) of the eligible persons opted not to enter in the prize drawing. If they did not enter the raffle or were not one of the three prize winners chosen at random, then participation entailed receiving no other rewards/incentives/remunerations.

Extremely detailed information about the study, its content, its initial development, and its implementation may be found in James et al. (2016). The original USTS2015 study received institutional review board approval from the University of California–Los Angeles prior to implementation. The present research for the secondary analysis of the USTS2015 data received institutional review board approval from California State University–Long Beach.

### 2.2. Measures Used

*Alcohol use classifications—**Nondrinkers*** are defined as people who reported never having consumed alcohol during their lifetime, or alternatively, those who have consumed alcohol during their lifetime but who reported no alcohol consumption during the 30 days prior to interview. ***Binge drinkers*** are defined as people who consumed five or more drinks in a short (i.e., few hours or less) period of time and who did so on fewer than four occasions (i.e., less often than weekly, on average) during the preceding month. Although the definition of binge drinking is usually adjusted by gender, with more alcohol consumption required of males in order to be considered binge drinkers than it is of females (typically five drinks for males versus four drinks for females; National Institute on Alcohol Abuse and Alcoholism, 2024), in the present study the higher threshold has been used for all respondents. This decision was made strategically because there is no way to know precisely “how far along” transgender individuals are in their medical transitioning. By selecting the higher threshold, the present authors have adopted a definition of binge drinking that is more conservative—one that is either accurate or an underestimation of the prevalence of binge drinking in this population. In accordance with the definitional criteria established by the National Institute on Alcohol Abuse and Alcoholism (2024), ***heavy drinkers*** are defined as persons who engaged in binge drinking four or more times during the month prior to the interview. It should be noted that, ordinarily, there is another way that someone might be classified as a heavy drinker—namely, if that individual consumed eight or more drinks per week (if female) or fifteen or more drinks per week (if male). However, in the NTS2015, this type of quantity–frequency information was not collected for non-binge style drinking. As a result, that additional consumption-based way of assigning people to the heavy drinking category was not an option in the present study. In this study, ***normal drinkers*** (for lack of a better way of referring to them) are those people who reported at least some alcohol use during the preceding month but who did not meet the definitions just listed for binge drinking or heavy drinking.

*Minority stress*—The principal variable examining minority stress in this study is a measure assessing the extent to which people experienced anti-transgender discrimination, harassment, and/or violence during the preceding year. This is a scale measure comprised of 20 items, each scored 0 (did not happen) or 1 (happened). These were: (1) low level of support from one’s family members for being transgender, (2) leaving or being ejected from a religious/faith community due to being transgender, (3) experiencing transgender-related discrimination or problems with one’s health insurance company, (4) experiencing discrimination, harassment, or substandard care from a doctor or other healthcare professional because of being transgender, (5) a general perception of being treated unequally as a result of being transgender, (6) experiencing verbal harassment from others due to being transgender, (7) being physically attacked by another person due to being transgender, (8) being harassed or threatened when using a public restroom, (9) termination from a job due to being transgender, (10) being forced or feeling coerced to leave a job due to being transgender, (11) not being hired for a job or not being promoted as a result of being transgender, (12) feeling a need to take specific steps at work in order to avoid transgender-related problems or confrontations, (13) having problems with one’s work supervisor as a result of being transgender, (14) being physically assaulted or attacked at work due to being transgender, (15) experiencing housing-related discrimination or harassment due to one’s gender identity or gender expression, (16) feeling a need to avoid utilizing public services just to minimize the chances of experiencing transgender-related discrimination or harassment, (17) experiencing bullying or other types of transgender-related harassment in school prior to high school graduation, (18) experiencing bullying or other types of transgender-related harassment in college, (19) being treated unequally or harassed by Transportation Security Administration (TSA) personnel when trying to travel, and (20) being treated unequally or harassed by members of the police force as a result of being transgender. This scale is reliable, with a Cronbach’s alpha coefficient of 0.76.

*Transition milestones*—In other published research (Klein & Washington, 2023b, 2023c), the present authors have demonstrated the importance of achieving versus not achieving various transition milestones^2^^,^^3^ when it comes to transgender persons’ mental health and behavioral outcomes. Accordingly, several such measures were included in the multivariate analyses in the present research as potential/likely covariates. These were: (1) the point at which the person had told any of his/her/their family members that he/she/they is/are transgender, (2) the point at which the person had told any of his/her/their friends that he/she/they is/are transgender, (3) the point at which the person had told any of his/her/their coworkers, supervisors, classmates, or others at school about being transgender^4^, (4) changing one’s name or gender on at least one form of official identification (e.g., Social Security card, driver’s license, student records, birth certificate, passport, etc.), (5) having begun taking hormones to facilitate the physical transitioning of genders, and (6) having undergone any gender-affirming surgical procedure. Each milestone was scored as 0 (not reached) or 1 (milestone reached).

For the present paper’s multivariate analyses, two *dependent variables* were examined: overall level of psychological distress and suicidal ideation. Respondents’ overall level of psychological distress during the previous 30 days was assessed using the Kessler-6 Scale (Kessler et al., 2002). It consists of six items, summed for the purpose of creating the overall level of psychological distress scale, with ordinal responses including “never” (scored 0), “a little of the time” (scored 1), “some of the time” (scored 2), “most of the time” (scored 3), and “all of the time” (scored 4). Each item inquired how frequently, during the previous thirty days, people felt (1) so sad that nothing could cheer them up, (2) nervous, (3) restless or fidgety, (4) hopeless, (5) that everything was an effort, and (6) worthless. The scale is reliable, with a Cronbach’s alpha of 0.91. The other dependent variable of interest in these analyses, suicidal ideation, was assessed via a dichotomous measure indicating if the person had or had not thought about ending his/her/their life during the previous year.

Numerous *independent variables* were included as potential covariates in the multivariate analyses. Several of these were demographic-type variables: gender identity (dichotomous, transgender male or transgender female; persons who self-identified as nonbinary with no inclination toward the male or female gender were excluded from this particular measure), self-identification as binary or nonbinary (dichotomous), age (continuous), (3) race (dichotomous, comparing Caucasians to persons of color), relationship status (dichotomous, married/“partnered” versus not “involved”), (5) living near or below poverty line (yes/no), educational attainment (dichotomous, at least a college graduate versus persons with less education), unemployment status (dichotomous, unemployed versus not unemployed), and self-labeled visual conformity with one’s affirmed gender (i.e., ability to “pass” as a member of one’s gender of identity; coded yes/no).

### 2.3. Statistical Analysis

Throughout this paper, as a result of the large sample size, results are reported as being statistically significant whenever *p* < 0.01 instead of the usual *p* < 0.05. Using a more rigorous standard for construing a finding as being indicative of statistical significance is advisable in a study such as the present one, in which a large sample size is used. The use of this level of increased scientific rigor is supported by statisticians who have discussed the merits and drawbacks of adopting various *p*-value thresholds (Kim & Choi, 2021; Zhu, 2016).

For the first part of the analysis, the focus is on whether experiences with anti-transgender discrimination, harassment, and violence is related to alcohol use classification as a normal drinker, a binge drinker, or a heavy drinker. Analysis of variance was used to examine this question, with post hoc paired-comparisons tests being performed to determine which, if any, of the drinker classification groups differed from which other(s). As an additional way of presenting the data and performing a “deeper dive” into what the data show with regard to the relationship between alcohol use classification and anti-transgender experiences, Student’s *t* tests were also performed to compare anti-transgender experiences between (1) normal drinkers versus people who were binge or heavy drinkers, (2) heavy drinkers versus people who were normal or binge drinkers, and (3) nondrinkers versus heavy drinkers (omitting from analysis people who used alcohol “normally” or in the form of binge consumption).

Multivariate analyses were undertaken next to determine whether anti-transgender experiences were a relevant variable in understanding people’s classification as a normal drinker (versus individuals whose alcohol use exceeded the normal drinker threshold), a heavy drinker (versus individuals whose alcohol use did not reach the heavy drinker threshold), or a nondrinker versus a heavy drinker. Because the dependent variable in each of these analyses was a dichotomous measure, multivariate logistic regression was used. Initially, all covariates were entered into each equation alongside the anti-transgender scale measure. Then, nonsignificant predictors were removed in a stepwise fashion (to minimize the “statistical noise” that comes with co-entering numerous measures into the multivariate equation simultaneously) until a best-fit final model for each equation had been developed, consisting solely of statistically significant contributors.

In the final part of the analysis, the present authors wanted to determine whether anti-transgender experiences and alcohol use classification both contributed to an overall understanding of the likelihood that transgender people would experience psychological distress and/or suicidal ideation. For the psychological distress analysis, multiple regression was used; for the suicidal ideation analysis, multivariate logistic regression was used. Once again, for each analysis, both of the main measures of interest (i.e., anti-transgender experiences, alcohol use classification) were entered simultaneously with all of the covariates. Then, nonsignificant contributors were removed in a stepwise fashion until a best-fit final model for each equation was developed.

## 3. Results

### 3.1. Alcohol Use Classifications

Approximately one person in ten reported never having had a drink of alcohol in his/her/their lifetime (10.3%). An additional 27.6% of the study participants reported having had alcohol at some point during their lifetime but not during the month prior to the interview. Thus, in the present study, 37.9% of the respondents are considered nondrinkers (*n* = 10,598). Among those who reported alcohol use during the month prior to the interview, based on the criteria described above, 59.9% were deemed normal drinkers (*n* = 10,246), 26.4% were classified as binge drinkers (*n* = 4516), and 13.8% were classified as heavy drinkers (*n* = 2355).

### 3.2. Anti-Transgender Experiences and Alcohol Use Classification

Table 1 presents information about the frequency of each type of anti-transgender experience included in this study. As the table shows, the most commonly reported anti-transgender experiences were feeling a need to take proactive steps at work in order to avoid confrontations or problems as a result of being transgender (57.2%), having no family support or very low levels of family support for one’s transgender identity (54.4%), being verbally harassed because of being transgender (48.2%), being physically attacked as a result of being transgender (29.5%), and experiencing problems or mistreatment from a physician or other healthcare professional as a result of being transgender (28.8%). Overall, very few respondents (8.2%) reported experiencing none of the types of anti-transgender problems under study. Conversely, most people (55.1%) said that they had experienced four or more of the twenty problems included in this research.

**Figure 1:**
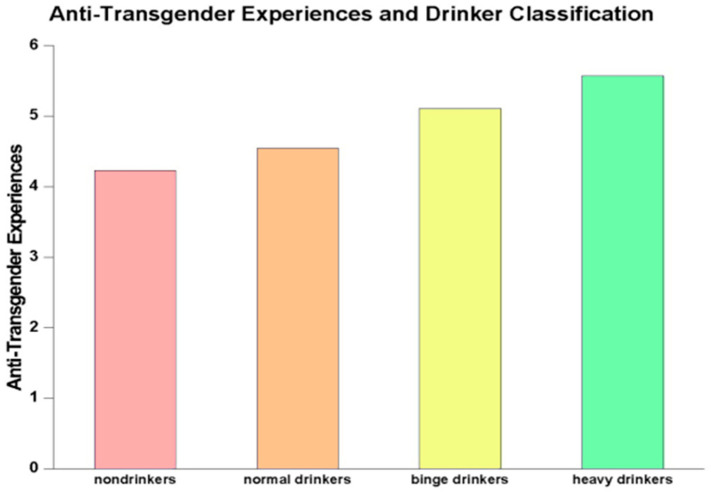
Anti-transgender experiences and drinker classification.

Figure 1 depicts the relationship between anti-transgender experiences and alcohol use classification. As the figure clearly shows, the more anti-transgender experiences people had incurred during the previous year, the more likely they were to engage in heavy drinking. Nondrinkers reported the fewest anti-transgender experiences with discrimination, harassment, and violence (mean = 4.23, SD = 3.39), followed by normal drinkers (mean = 4.55, SD = 3.40), binge drinkers (mean = 5.08, SD = 3.49), and heavy drinkers (mean = 5.52, SD = 3.83). This relationship was statistically significant (*p* < 0.0001).

**Figure 2:**
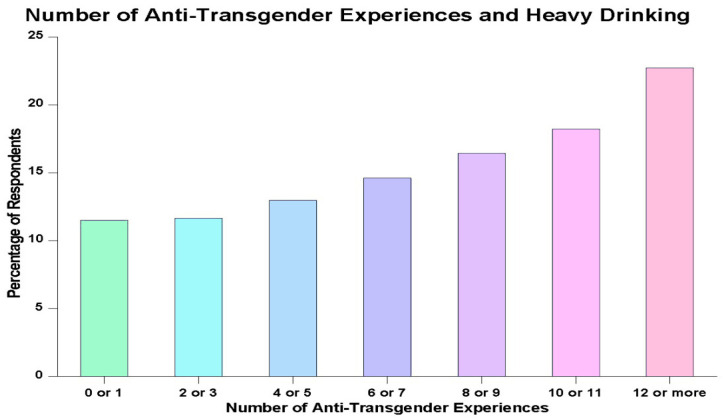
Number of anti-transgender experiences and heavy drinking.

Figure 2 shows the relationship between the number of anti-transgender experiences people reported with harassment, discrimination, and/or violence and the likelihood that they would be classified as a heavy drinker. The data clearly demonstrate that a greater number of anti-transgender experiences corresponds with an increased likelihood of being a heavy drinker. The differences are particularly noticeable when one compares the people who experienced the greatest number of anti-transgender problems with harassment, discrimination, and/or violence and those who experienced the fewest such problems. The former were nearly twice as likely as the latter to be classified as heavy drinkers (*p* < 0.0001).

People classified as normal drinkers reported, on average, 14.9% fewer anti-transgender experiences than their peers who were classified as binge drinkers or heavy drinkers (4.55 versus 5.23; *t* = 12.57, *p* < 0.0001). Similarly, heavy drinkers reported incurring 17.2% more anti-transgender experiences than their normal drinker and binge drinker counterparts (5.52 versus 4.71, *t* = 10.43, *p* < 0.0001). The intergroup differences are even more stark when nondrinkers and heavy drinkers are compared, with the latter reporting 30.5% more problems with anti-transgender discrimination, harassment, and violence than the former (4.23 versus 5.52, *t* = 16.31, *p* < 0.0001).

### 3.3. Multivariate Analyses

Table 2 presents the results of the multivariate analysis comparing people classified as normal drinkers to people classified as binge drinkers or heavy drinkers. The multivariate model did retain the anti-transgender experiences measure among the final group of seven significant predictors that differentiated people classified as normal drinkers from those classified as binge drinkers or heavy drinkers (*p* < 0.0001). Anti-transgender experiences were retained in this final model along with gender (*p* = 0.001), age (*p* < 0.0001), relationship status (*p* = 0.0021), nonbinary self-identification (*p* < 0.0001), the transition milestone pertaining to disclosing one’s transgender identity to family members (*p* < 0.0001), and the transition milestone pertaining to gender-conforming surgical procedures (*p* < 0.0001). Anti-transgender experiences were the second most consequential predictor in the model (*p* < 0.0001), ranking only behind age in its importance.

Table 3 presents the results of the multivariate analysis comparing people classified as heavy drinkers to people classified as normal drinkers or binge drinkers. The table shows that anti-transgender experiences were the single most important variable in the final multivariate model (*p* < 0.0001), with nearly twice the explanatory power as the next-most-important measure in the equation. Anti-transgender experiences were retained in the model, along with gender identity (*p* = 0.0036), nonbinary self-identification (*p* < 0.0001), and two of the transition milestones (namely, having disclosed one’s transgender identity to people at school or work [*p* = 0.001] and having changed one’s name and/or gender on legal documents [*p* < 0.0001].

Table 4 presents the results of the multivariate analysis comparing people classified as nondrinkers to people classified as heavy drinkers. Four measures were retained in the final equation: anti-transgender experiences (*p* < 0.0001), gender identity (*p* < 0.0001), educational attainment (*p* < 0.0001), and poverty (*p* < 0.0001). Of these, anti-transgender experiences were the strongest differentiator between nondrinkers and heavy drinkers.

Table 5 presents the findings for the multivariate analysis examining the roles played by anti-transgender experiences and alcohol use classification in the prediction of overall levels of psychological distress. In this model, which explained 28.7% of the total variance, both anti-transgender experiences and alcohol use classification were retained as significant contributors (*p* < 0.0001 for both). They appear in the equation alongside ten other unique contributors (all of which were *p* < 0.0001): gender identity, age, educational attainment, poverty, nonbinary self-identification, the transition milestones pertaining to disclosure of one’s transgender identity to family, coworkers, or classmates, the transition milestone pertaining to changing one’s name and/or gender on official legal documents, the transition milestone of taking gender-affirming hormones, and the transition milestone pertaining to gender-affirming surgical procedures. In this multivariate equation, alcohol use classification and age were by far the most consequential of the independent variables retained.

Table 6 presents the findings for the multivariate analysis pertaining to the roles played by anti-transgender experiences and alcohol use classification in the prediction of whether or not people had thought about dying by suicide during the preceding year. Both measures were found to be strongly (*p* < 0.0001) predictive of whether or not people had thought about terminating their lives. In fact, alcohol use classification was by far the single strongest predictor in the model. Also contributing to the differentiation between people who had and those who had not thought about dying by suicide during the preceding year were gender identity, age, educational attainment, relationship status, poverty, and four of the transition milestones (all were *p* < 0.0001).

## 4. Discussion

This study has shown that heavy drinking is fairly commonplace among transgender adults. Among individuals reporting any alcohol use during the preceding month, nearly 1 in 7 met the definition of heavy drinkers used in this study. It should be noted that this prevalence (13.8%) is a low-end, conservative estimate because the higher threshold for establishing eligibility as a problem drinker—usually reserved only for men—was applied to all respondents in this study. Not applying different threshold criteria for males and females was a strategic decision on the present researchers’ part, implemented in recognition of the fact that gender transitioning might be incomplete among some individuals, thereby rendering the “fifteen or drinks per week for males” and “eight or more per week drinks for females” criteria that are usually used to establish the eligibility to be classified as a heavy drinker to be considered dubious or, potentially, inappropriate/inapplicable. Even using the more stringent definition of heavy drinking, this study’s 13.8% prevalence rate is more than twice the national average, usually cited as being 6% (Centers for Disease Control and Prevention [CDC], 2024). Similarly, the present study’s finding regarding the prevalence of binge drinking among transgender adults (26.4%) is also considerably higher than that typically reported for the population-at-large, usually said to be approximately 17% (Centers for Disease Control and Prevention [CDC], 2024). (The present authors would like to point out that recent evidence suggests that the rates of heavy drinking and binge drinking rose sharply during the years of the COVID-19 pandemic—by approximately 20% according to very recent statistics—and have remained elevated since the worst of the pandemic began to wind down (Somayajula et al., 2024)). Thus, not only are the present study’s prevalence estimates conservative by virtue of the operational definition chosen for use in this study, but in all likelihood they are even more conservative than what is being reported in the population-at-large as a result of the sharp increases observed since 2020. These findings are in line with those reported by previous researchers, who have also noted that heavy drinking and binge drinking are significantly more prevalent among members of the lesbian, gay, bisexual, transgender, and queer (LGBTQ) community than among their heterosexual counterparts (Gitelman et al., 2024; Shokoohi et al., 2022). Higher reported rates of heavy and/or binge drinking have also been reported specifically for transgender persons when they are compared to people who do not self-identify as transgender (Gilbert et al., 2018; Lehavot et al., 2022). The high prevalence of both heavy drinking and binge drinking identified in the present study and by previous researchers highlights the need to understand better the factors underlying these adverse drinking practices.

It is in that very vein that the present study examined respondents’ experiences with anti-transgender discrimination, harassment, and/or violence; and the present authors treated such experiences as a proxy measure for minority stress. The large majority of respondents in this study (92.0%) reported having experienced at least one type of anti-transgender discrimination, harassment, or violence under study during the year prior to the interview. Most (50.3%), in fact, reported having had at least four such experiences, with more than 1 person in 10 (10.1%) reporting having had ten or more such experiences. The mean number of different types of anti-transgender experiences incurred during the previous year was 4.82 (*SD* = 3.51). Simply put, these types of minority stressor occurrences are highly prevalent in the lives of most transgender adults in the United States. Other researchers, too, have reported on the high prevalence of various types of discriminatory, harassing, and violent experiences in transgender persons’ lives (Sherman et al., 2020; Stenersen et al., 2022; Tucker et al., 2019).

Not surprisingly, such experiences with anti-transgender discrimination, harassment, and violence have been linked with a wide variety of negative outcomes. Included among these are a heightened risk of depression (Kimber et al., 2024; White Hughto & Reisner, 2018; Wilson et al., 2024; Wolford-Clevenger et al., 2021), higher odds of thinking about dying by suicide (Meza Lazaro & Bacio, 2023; Pletta et al., 2024; Trujillo et al., 2017), an elevated risk of experiencing anxiety (Kimber et al., 2024; Klemmer et al., 2021; Wilson et al., 2024), an increased risk of using illegal drugs (Meza Lazaro & Bacio, 2023; Wolford-Clevenger et al., 2021), a higher prevalence of eating disorders and disordered eating behaviors (Cusack et al., 2021; Drabish & Theeke, 2022; Urban et al., 2024), and a host of other adverse outcomes.

In the present study, the researchers were interested specifically in the nexus of experiencing minority stressors and heavy drinking. The results obtained were very clear: The more anti-transgender experiences people reported, the more likely they were to engage in heavy drinking. Nowhere was this more evident than in the data presented in Figure 2, where there was nearly a nearly doubled risk of engaging in heavy drinking when people were in the group reporting the greatest number of anti-transgender experiences compared to those who were in the group reporting either no such experiences or only one such experience. The multivariate analyses conducted as part of the present study buttressed this finding even more by demonstrating that the relationship between anti-transgender experiences and heavy drinking is a robust one. It was sustained even when the impact of numerous other potentially confounding variables was taken into account. Moreover, this proxy measure of minority stress was retained in all of the models examined. In most of them, it was either the single most impactful measure predicting heavy drinking or the second most impactful measure.

Unmistakably, this study has demonstrated that minority stressors—as measured here by the number of different types of experiences people had with anti-transgender discrimination, harassment, and/or violence—are a strong predictor of heavy drinking among transgender adults. This study also found the relationship almost to be a dose–response type of relationship. This is very similar to the results reported by Tran et al. (2023) in their study of the relationship between minority stress and hazardous drinking among transgender adults. This finding is also supportive of the concept of allostatic load (described earlier in this paper), in which repeated exposure to adverse conditions (such as the types of anti-transgender discrimination, harassment, and violence examined in the present study) leads to greater and greater adverse impact. It highlights the crucial need to find ways to reduce the prevalence of anti-transgender experiences in the daily lives of transgender individuals and demonstrates one of the many risks (here, being classified as a heavy drinker) inherent in continually experiencing problems with discrimination, harassment, and violence. Other researchers have written about the relationship between minority stressors and various types of unhealthy alcohol use behaviors and drinking patterns among transgender persons (Gonzalez et al., 2017; Lindley et al., 2021; Mammadli et al., 2023; Meza Lazaro & Bacio, 2023), and they have almost unanimously concluded that effective strategies to combat anti-transgender experiences are needed in order to improve this aspect of transgender persons’ lives. The present study stands in strong support of their work in this area.

One final thing that the present authors would like to point out is that this study has focused specifically on heavy drinking. As mentioned earlier, most of the published research on the topic of alcohol use/abuse among transgender persons has focused either on hazardous drinking (as typically measured via the AUDIT) or on binge drinking, with very little attention having been devoted previously to heavy drinking specifically. Thus, one of the present study’s key contributions to the scientific literature lies in its focus on heavy drinking. This is important because it represents a level of drinking—both a quantity and a periodicity to the alcohol consumption—that is likely to be more serious and more problematic than “mere” hazardous drinking or binge drinking classifications that have been examined and discussed more thoroughly in the scientific literature (First Step Behavioral Health, 2024; SMART Recovery, 2024). This makes the present study’s finding regarding the nexus between anti-transgender experiences/minority stress and heavy drinking even more important, because the consequences of those anti-transgender experiences extend not only to their direct impact on the mental health of affected persons but also to behavioral outcomes (such as heavy drinking) that are likely to be particularly dangerous in their lives.

*Limitations of This Research*—As with any study, there are numerous potential limitations inherent in this study. First, the data relied upon uncorroborated self-reported behaviors. Although a common practice in social science research, it is nonetheless a potential source of inaccuracy in the data. Coinciding with this, the USTS2015 did not entail probability sampling and, thus, is subject to issues pertaining to the representativeness of the sample when compared to the population at large. Feldman et al. (2021), however, analyzed the population characteristics data from this study and compared it to both the TransPop Study and the U.S. population at large and reported that the USTS2015 research sample closely matches the nation as a whole on a variety of health-related outcomes such as those used/reported in the present study. Thus, the generalizability of the findings from this research is likely to be fairly good. Third, the data were collected in 2015 and are not as recent as would be considered ideal. In the context of drinking practices, however, this is not likely to be a major problem because these behaviors are generally considered to be reasonably stable over time. Fourth, because of the nature of gender transitioning among transgender individuals and the fact that different respondents were at different points along the gender transitioning continuum, this study relied upon a definition of heavy drinking that was more restrictive than usual. The authors deemed this prudent, though, so that there would be no accusations of overstating the main problem/phenomenon under study. Fifth, the use of multiple regression as the analytic approach to examining the data brings with it assumptions regarding which variables are likely to be causal/underlying to which others. Causal ordering of variables is analyzed more powerfully with longitudinal data than it is with cross-sectional data, but it is commonplace for researchers to rely upon their research hypotheses and findings from previous studies to guide assumptions regarding the variables included in these models. Finally, any number of additional variables could have been examined as potential independent/confounding variables in the multivariate analyses. The present authors selected those that are either “standard inclusions” in social science research (e.g., age, educational attainment, etc.) or measures that have been shown in their own previous research focusing on transgender individuals (e.g., transition milestones, visual conformity with one’s gender identity) to be relevant predictors. Nonetheless, numerous other measures could have been examined had such data been available to the researchers.

## Figures and Tables

**Table 1:** Frequency of each type of anti-transgender experience reported.

Type of Anti-Transgender Experience	Prevalence (%)
Having no family support or very low level of family support due to being transgender	54.4
Leaving a faith community or being ejected from a faith community due to being transgender	26.6
Experiencing problems or discrimination from one’s health insurance company due to being transgender	24.0
Experiencing problems or mistreatment from doctors or other healthcare professionals due to being transgender	28.8
Perceiving oneself to receive unequal treatment from others due to being transgender	13.8
Being verbally harassed because of being transgender	48.2
Being physically attacked because of being transgender	9.0
Being verbally harassed or threatened when using a public restroom	25.9
Being terminated from a job due to being transgender	11.7
Being forced to resign or leave a job due to being transgender	10.0
Not being hired for a job or not being promoted at work due to being transgender	22.4
Feeling a need to take precautionary actions at work in order to avoid confrontations with coworkers or supervisors	57.2
Experiencing problems with one’s supervisor at work due to being transgender	16.8
Being physically assaulted or attacked at work due to being transgender	11.1
Experiencing housing-related problems or discrimination due to being transgender	21.4
Feeling a need to avoid using publicly available services in order to avoid problems or confrontations resulting from being transgender	18.8
Experiencing problems or harassment at school prior to high school graduation as a result of being transgender	29.5
Experiencing problems or harassment at college as a result of being transgender	8.9
Experiencing problems, harassment, or mistreatment from TSA officials when trying to travel	22.9
Experiencing problems, harassment, or mistreatment from police officials as a result of being transgender	10.3
	
None of these problems	8.2
One or two of these problems	23.4
Three or four of these problems	26.1
Five or six of these problems	17.6
Seven or eight of these problems	11.2
Nine or more of these problems	13.5

**Table 2:** Multivariate analysis for anti-transgender experiences and alcohol use classification: normal drinkers versus binge or heavy drinkers.

Independent Variable	b	β	*p* < |x|
Anti-Transgender Experiences	−0.05	0.10	0.0001
Gender Identity = Female	−0.12	0.03	0.0010
Age	0.02	0.16	0.0001
Relationship Status: “Involved”	0.14	0.03	0.0021
Nonbinary	0.19	0.05	0.0001
Transition Milestone: Told Family Members about Being Transgender	0.19	0.04	0.0001
Transition Milestone: Had Any Gender-Conforming Surgery	0.16	0.04	0.0001

**Table 3:** Multivariate analysis for anti-transgender experiences and alcohol use classification: heavy drinkers versus normal or binge drinkers.

Independent Variable	b	β	*p* < |x|
Anti-Transgender Experiences	0.07	0.13	0.0001
Gender Identity = Female	0.15	0.04	0.0036
Nonbinary	−0.25	0.07	0.0001
Transition Milestone: Told Coworkers or Classmates About Being Transgender	−0.18	0.05	0.0010
Transition Milestone: Began Changing Name and/or Gender on Official Legal Documents	−0.21	0.06	0.0001

**Table 4:** Multivariate analysis for anti-transgender experiences and alcohol use classification: nondrinkers versus heavy drinkers.

Independent Variable	b	β	*p* < |x|
Anti-Transgender Experiences	0.09	0.18	0.0001
Gender Identity = Female	0.20	0.05	0.0001
Educational Attainment = At Least College Graduate	0.62	0.16	0.0001
Poverty	−0.21	0.06	0.0001

**Table 5:** Multivariate analysis for level of psychological distress: the roles played by anti-transgender experiences and alcohol use classification.

Independent Variable	b	β	*p* < |x|
Anti-Transgender Experiences	0.46	0.07	0.0001
Alcohol Use Classification	0.58	0.27	0.0001
Gender Identity = Female	0.51	0.04	0.0001
Age	−0.12	0.25	0.0001
Educational Attainment = At Least College Graduate	−1.23	0.11	0.0001
Poverty	0.80	0.06	0.0001
Nonbinary Self-Identification	0.47	0.04	0.0001
Transition Milestone: Disclosure of Transgender Identity to Family Members	−0.52	0.04	0.0001
Transition Milestone: Disclosure of Transgender Identity to Classmates or Coworkers	−1.05	0.08	0.0001
Transition Milestone: Changing Name and/or Gender on Official Legal Documents	−1.11	0.09	0.0001
Transition Milestone: Taking Gender-Affirming Hormones	−1.12	0.10	0.0001
Transition Milestone: Undergoing Gender-Affirming Surgical Procedures	−0.51	0.04	0.0001

**Table 6:** Multivariate analysis for suicidal ideation: the roles played by anti-transgender experiences and alcohol use classification.

Independent Variable	OR (95% CI)	β	*p* < |x|
Anti-Transgender Experiences	1.18 (1.16–1.19)	0.05	0.0001
Alcohol Use Classification	1.15 (1.15–1.21)	0.31	0.0001
Gender Identity = Female	1.34 (1.23–1.45)	0.08	0.0001
Age	0.97 (0.97–0.98)	0.19	0.0001
Educational Attainment = At Least College Graduate	0.65 (0.60–0.70)	0.12	0.0001
Relationship Status = “Involved”	0.84 (0.76–0.93)	0.04	0.0011
Poverty	1.23 (1.13–1.34)	0.05	0.0001
Transition Milestone: Disclosure of Transgender Identity to Classmates or Coworkers	0.77 (0.71–0.84)	0.07	0.0001
Transition Milestone: Changing Name and/or Gender on Official Legal Documents	0.76 (0.69–0.83)	0.08	0.0001
Transition Milestone: Taking Gender-Affirming Hormones	0.80 (0.73–0.89)	0.06	0.0001
Transition Milestone: Undergoing Gender-Affirming Surgical Procedures	0.84 (0.76–0.92)	0.05	0.0004

## Data Availability

Due to data security issues and privacy concerns, the data used in the preparation of this paper cannot be made available publicly.

## Notes

1 For readers unfamiliar with the term *transition milestones*, these may be thought of as specific events or occurrences in transgender persons’ transitioning experiences that demarcate their lives in a “before X happened” versus “after X happened” kind of way. These transition milestones represent different stages of progress in transgender persons’ journey toward fully accepting their gender identity and incorporating that identity more fully into their everyday lives. Each milestone, once reached or achieved, is one additional step toward living authentically and completely as a member of the gender with which the person identifies most closely. Across these various studies, a wide array of measures have been used to assess allostatic load, making it somewhat difficult and unwise to try to compare “apples to apples”. For example, DuBois and Juster (2022, p. 6) examined 10 biomarkers representing neuroendocrine (cortisol awakening response and systemic HPA-axis output from mid-morning until bedtime), immune (C-reactive protein, Epstein–Barr virus antibodies), cardiovascular (heart rate, systolic and diastolic blood pressure), and metabolic (body mass index (BMI), percent body fat, and triceps skinfold measurement) functioning. In contrast, Ferina (2024) spoke of a variety of experiences as factors contributing to the allostatic load burdening the respondents in her research. These included, among others, relocating to a new area, transferring to a new school, the pressure to perform well in school, feeling a need to excel in athletic activities, experiencing prejudice and/or discrimination based on one’s demographic characteristics, romantic relationship instability, and grappling with gender identity. Based on their review of the literature pertaining to allostatic load and health outcomes, Guidi et al. (2021) wrote about the myriad ways that allostatic load has been defined and studied in the scholarly literature. These authors specifically mentioned that sometimes, allostatic load has been studied using biological markers whereas, at other times, it has been examined via what they termed ‘clinimetrics’—ostensibly, clinical measurements of psychological and/or psychosocial distress. Truly, there has been no consensus or consistency in the published literature regarding the way(s) that allostatic load can be measured.

2 For readers unfamiliar with the term *transition milestones*, these may be thought of as specific events or occurrences in transgender persons’ transitioning experiences that demarcate their lives in a “before X happened” versus “after X happened” kind of way. These transition milestones represent different stages of progress in transgender persons’ journey toward fully accepting their gender identity and incorporating that identity more fully into their everyday lives. Each milestone, once reached or achieved, is one additional step toward living authentically and completely as a member of the gender with which the person identifies most closely.

3 The authors would like to point out that it is important to bear in mind that not all transgender persons will choose to pursue each and every transition milestone. For example, many transgender individuals have no interest in undergoing gender-affirming surgical procedures. Others opt to eschew the use of gender-affirming hormones, either because they are uninterested in taking such medications and/or because they have had bad previous experiences when taking such medications. Yet other transgender persons might not want to inform family members or coworkers (or other people in their life) that they are transgender. In the present research, transition milestones are included as a covariate because previous research—both by the present investigators and numerous other authors—has shown that attaining these milestones oftentimes is linked with various behavioral and mental health outcomes. Research has demonstrated that the benefits derived from reaching these milestones are numerous. Thus, their impact ought to be considered whenever possible (as is the case in the present study). No value judgment whatsoever is intended by the authors regarding whether or not people choose to pursue specific transition milestones. The inclusion of these measures in the present study is the direct result of the myriad research findings indicating their relevance to positive behavioral and mental health outcomes among transgender persons.

4 Persons who were not enrolled in school, and those who were either unemployed or unable to work due to a disability, were excluded from consideration in this particular transition milestone.
